# Long-Term Functional Consequences and Ongoing Cerebral Inflammation after Subarachnoid Hemorrhage in the Rat

**DOI:** 10.1371/journal.pone.0090584

**Published:** 2014-03-06

**Authors:** Elke Kooijman, Cora H. Nijboer, Cindy T. J. van Velthoven, Wouter Mol, Rick M. Dijkhuizen, Jozef Kesecioglu, Cobi J. Heijnen

**Affiliations:** 1 Department of Intensive Care Medicine, University Medical Center Utrecht, Utrecht, The Netherlands; 2 Laboratory of Neuroimmunology and Developmental Origins of Disease, University Medical Center Utrecht, Utrecht, The Netherlands; 3 Image Sciences Institute, University Medical Center Utrecht, Utrecht, The Netherlands; 4 Department of Symptom Research, Division of Internal Medicine, The University of Texas MD Anderson Cancer Center, Houston, Texas, United States of America; Hôpital Robert Debré, France

## Abstract

Subarachnoid hemorrhage (SAH) represents a considerable health problem with an incidence of 6–7 per 100.000 individuals per year in Western society. We investigated the long-term consequences of SAH on behavior, neuroinflammation and gray- and white-matter damage using an endovascular puncture model in Wistar rats. Rats were divided into a mild or severe SAH group based on their acute neurological score at 24 h post-SAH. The degree of hemorrhage determined in post-mortem brains at 48 h strongly correlated with the acute neurological score. Severe SAH induced increased TNF-α, IL-1β, IL-10, MCP-1, MIP2, CINC-1 mRNA expression and cortical neutrophil influx at 48 h post-insult. Neuroinflammation after SAH was very long-lasting and still present at day 21 as determined by Iba-1 staining (microglia/macrophages) and GFAP (astrocytes). Long-term neuroinflammation was strongly associated with the degree of severity of SAH. Cerebral damage to gray- and white-matter was visualized by immunohistochemistry for MAP2 and MBP at 21 days after SAH. Severe SAH induced significant gray- and white-matter damage. MAP2 loss at day 21 correlated significantly with the acute neurological score determined at 24 h post-SAH. Sensorimotor behavior, determined by the adhesive removal task and von Frey test, was affected after severe SAH at day 21. In conclusion, we are the first to show that SAH induces ongoing cortical inflammation. Moreover, SAH induces mainly cortical long-term brain damage, which is associated with long-term sensorimotor damage.

## Introduction

Subarachnoid hemorrhage (SAH) results from the rupture of an aneurysm and a subsequent flow of blood into the subarachnoid space of the brain. In humans SAH has a mortality rate of approximately 40–50% and represents a considerable public health problem. In general, surviving patients are confronted with a great loss of quality of life. Furthermore, the consequences of SAH represent an economic burden for society since 50% of the patients are below 55 years [1], [2]. Directly after SAH, several functional changes occur, including an increase in cerebral blood pressure (CBP), intracranial pressure (ICP) and mean arterial blood pressure (MBP). More delayed secondary processes like micro-thrombo-emboli formation, cerebral edema formation and reduced cerebral perfusion or delayed cerebral ischemia (DCI) may also contribute to exacerbating SAH brain damage. DCI, also referred to as delayed ischemic neurological deficit, is a poorly understood complication of SAH in patients. The diagnosis of DCI is complex; laboratory examinations and brain imaging are needed to exclude other causes [3]. Cerebral vasospasm is thought to be an important underlying mechanism of the development of DCI. In humans, vasospasms are most prominent near the site of the aneurysm [4]. So, brain damage after SAH is multi-factorial; after the initial damage, secondary brain injury is considered to be a major contributor to increased mortality and morbidity after SAH [2], [5], [6]. The interventions currently applied have a short time window and should be applied as soon as possible after SAH. These current treatments include calcium antagonists, triple-H therapy and surgery directly after the hemorrhage to prevent rebleeding [7], [8]. Considering the relatively young age of the patients, there is a great need for efficacious novel therapeutic interventions with a longer time-window of treatment. To explore the efficacy of new therapeutic targets, however, a well-validated animal model of SAH is needed. Currently, two animal models mimicking SAH are frequently used: 1) injection of autologous blood in the brain (one or multiple injections) or 2) perforation of the middle cerebral artery (MCA) at the level of the circle of Willis (the endovascular puncture model). The endovascular puncture model most closely resembles human SAH but long-term follow-up data are very limited [5]–[8]. The model using injection of autologous blood is more frequently used to study SAH since it has a low mortality rate. In this model, it has been shown that neuroinflammation starts within 3–6 hours after the injection of autologous blood and lasts for approximately 2 days [9]–[11]. Importantly, the long-term effects of SAH on neuroinflammation and damage are not known, as to the best of our knowledge in all studies, animals were terminated within a couple of days to maximum of a week, except for 3 studies in which the animals survived for 14 or 28 days post-SAH [12]–[14]. However, in these latter long-term studies, the focus was on behavioral changes and parameters like lesion size, inflammation and cell death were not determined [12]–[14]. A drawback of the blood injection model of SAH is that there is hardly any variation in SAH severity, since a fixed amount blood is injected. These former data together show the necessity of a long-term study investigating lesion size, cerebral inflammation and behavioral deficits after SAH in an experimental model which is more comparable to the clinical situation. The aim of this study is therefore to describe the long-term consequences of SAH using the endovascular puncture model with a special emphasis on neuroinflammation and gray- and white-matter loss. Moreover, we studied the long-term consequences of SAH on behavior focusing on fine sensorimotor behavior.

## Methods

### Animals

Experiments were performed in accordance with the Dutch regulations, the European international guidelines (Directive 86/609, ETS 123, Annex II) and approved by the Experimental Animal Committee of the University Medical Center Utrecht. Adult male Wistar rats (Charles River, Maastricht, The Netherlands) of 300–350 grams were housed in groups of 4 on reversed light/dark cycle with *ad libidum* food access. Animals were randomly assigned to 4 different experimental groups: SAH or sham-operation, the latter reflecting the entire procedure except for puncturing of the vessel. These 2 experimental groups were again randomly assigned to a 48 hours survival group and a 21 days survival group. We started with a total of 90 animals. 57 rats underwent SAH, of which 24 animals died or were euthanatized pretermly when reaching the humane endpoint (see below), 21 animals were designated to the mildly-affected SAH group and 12 animals to the severely-affected SAH group. 8 mildly-affected SAH animals were terminated at 48 h and 13 survived for 3 weeks. 6 severely-affected SAH animals were terminated at 48 h and 6 survived for 3 weeks. 33 animals underwent sham-operation of which 0 died, 14 rats were terminated at 48 h and 19 animals at 3 weeks post-SAH.

### Experimental subarachnoid hemorrhage

Rats were anesthetized by mechanical ventilation with 2% isoflurane in air/oxygen (2:1). An intramuscular injection of 5 mg/kg gentamicin (Centrafarm, Ettenleur, The Netherlands) was administered to prevent infections. Core temperature was maintained at 37.5°C using a temperature-controlled heating pad. SAH was induced as described previously [15]. In short, a sharpened 3.0 prolene suture was introduced into the right external carotid artery (ECA) and advanced through the internal carotid artery (ICA) while clamping the common carotid artery (CA) in front of the junction toward ICA and ECA. The suture was further advanced into the intracranial ICA until resistance was felt and then pushed 3 mm further penetrating the ICA near the bifurcation with the middle cerebral artery (MCA). The suture was then withdrawn, the ECA was closed entirely and the CA clamp was released which leads to reperfusion in the ICA and producing SAH. The duration of endovascular occlusion was 30 seconds to 2 minutes. Reperfusion occurred after 2–3 minutes. Sham-operated control rats underwent an identical procedure, however, without puncturing the bifurcation of the ICA and MCA.

To minimize suffering, animals received i.p. 30 µg/kg buprenorphine (Reckitt & Colman, Kingston-Upon-Hill, UK) for pain relief starting 30 min before the surgical procedure, which was repeated 3 times daily on day 1 and 2 post-SAH. After surgery, animals recovered in their home cage which was placed on a heating pad and animals had access to soak food *ad libidum*. Animals were monitored 2 times daily for well-being (see below at humane endpoint) and weight was measured daily for 7 days. Weight loss >10 g per 24 h was compensated by subcutaneous injection of 5 ml Ringer's lactate (Baxter, Utrecht, The Netherlands).

Humane endpoint was reached when weight loss was determined to be >20% or when one or more of the following behaviors were determined: piloerection, tremor, salivation, breathlessness/panting, unresponsiveness to stimuli and self-mutilation. Furthermore, the neurological score determined daily for 6 days post-SAH (see below) was used to determine humane endpoint. When the neurological function was 5 or lower, humane endpoint was reached.

### Grading system of SAH post-mortem

Photographs were taken from the base of the brain at 48 h after SAH (Canon Powershot SX200, camera). To score the extent of bleeding, a grid dividing the basal cistern in six segments was used to analyze the photographs. Each segment was scored from 0–3 depending on the amount of blood. Grade 0: no subarachnoid blood; grade 1: minimal subarachnoid blood; grade 2: moderate clot; grade 3: blood clot and recognizable arteries. Total score (0–18) was calculated as the sum of six segments.

### Real-time RT-PCR

Rats were terminated at 48 h post-SAH and cortices (non-perfused) were rapidly dissected on ice and frozen in liquid nitrogen. Cortices were pulverized on liquid nitrogen and aliquots were stored at −80C. RNA from 50–100 mg pulverized brain cortex was extracted using TRIzol® Reagents (Invitrogen, Paisley, UK). RNA concentration was determined using a spectrophotometer. cDNA was synthesized with SuperScript Reverse Transcriptase (Invitrogen). The PCR reaction was performed using SybrGreen with the I-cycler IQ5 Real-Time PCR Detection System (Bio-Rad). The following primers were used: Tumor necrosis factor α (TNFα) FW: 5′-CCCAGACCCTCACACTCAGATCAT-3′, REV:5′-CAGCCTTGTCCCTTGAAGAGAA-3′; Interleukin (IL) 1-β FW: 5′-CTCTGTGACTCGTGGGATGATG-3′, REV:5′-CACTTGTTGGCTTATGTTCTGTCC-3′; IL-10 FW: 5′-CCTTACTGCAGGACTTTAAGGGTTA-3′; REV: 5′-TTTCTGGGCCATGGTTCTCT-3′; Monocyte chemotactic protein-1 (MCP-1) FW: 5′-CAGAAACCAGCCAACTCTCA-3′, REV: 5′-GTGGGGCATTAACTGCATCT-3′; Macrophage inhibitory protein 2 (MIP2) FW: 5′-TTGTCTCAACCCTGAAGCCC-3′, REV:5′-TGCCCGTTGAGGTACAGGAG-3′; Chemokine induced neutrophil chemoattractant-1 (CINC-1) FW:5′-CCAAAAGATGCTAAAGGGTGTCC-3′, REV: ′5-CAGAAGCCAGCGTTCACCA-3′.

### Myeloperoxidase (MPO) Assay

MPO activity was determined as described previously [16]. In short, non-perfused pulverized brain cortex from rats terminated 48 h post-SAH was homogenized in HEPES buffer (50 mM, pH 8.0), centrifuged and pellet was homogenized in H_2_O/0.5%CTAC (Merck, Darmstadt, Germany). Following centrifugation, supernatants were diluted in 5 mM citrate buffer (pH 5.0)/0.22% CTAC. Substrate buffer, containing 3 mM 3′,5,5′ –tetramethylbenzidine dihydrochloride (Sigma-Aldrich, Steinheim, Germany), 120 µM resorcinol (Merck) and 2.2 mM H_2_O_2_, was added. Reaction mixtures were incubated for 30 minutes at 37°C and 4N H_2_SO_4_ was added to stop the reaction. Optical Density (OD) was determined at 450 nm. MPO extract from a known amount of neutrophils was used to obtain a reference curve.

### Histology

Rats were terminated at 21 days post-SAH by pentobarbital overdose and perfused with 4% paraformaldehyde. Brains were post-fixed, embedded in paraffin and coronal sections were cut. Deparaffinized sections were incubated with mouse-anti-microtubule-associated protein 2 (MAP2) (1∶1000; Sigma-Aldrich), mouse-anti-myelin basic protein (MBP) (1∶1600; Sternberger Monoclonals, Lutherville, MD), mouse-anti-glial fibrillary acidic protein (GFAP) (1∶200; Cymbus biotechnology, Southampton, UK), or rabbit-anti-ionized calcium binding adaptor molecule 1 (Iba-1) (1∶500; Wako, Germany) followed by biotin-labeled horse-anti-mouse or goat-anti-rabbit secondary antibodies. Staining was revealed using Vectastain ABC kit (Vector Laboratories, Burlingame, CA) and diaminobenzamidine. Full section images were captured by MAP2 and MBP staining. The Contra- and ipsilateral hemisphere was outlined manually, and the ratio of ipsi- to contralateral areas was calculated by using Adobe Photoshop (MAP2) and MacBiophotonics Imgae J (MBP) software [17]. Photographs of Iba-1 and GFAP sections were taken with an Axio lab A.1 microscope and AxioCam ICc5 camera (both Zeiss, Oberkochen, Germany). Quantification of Iba-1- and GFAP-positivity was determined in 5 cortical areas (Bregma 0.12 mm–1.56 mm) by measuring the Iba-1/GFAP-positive pixels/mm^2^ using MacBiophotonics ImageJ software. All measurements were performed by one investigator.

### Functional initial outcome

To monitor the initial consequences of surgery, we determined the acute neurological deficits before SAH (day 0) and at days 1 to 7 post-SAH by an adaptation of the method described by Sugawara et al.[18] In short we determined spontaneous activity, spontaneous movements of all limbs, movement of forelimbs (outstretching) while held by tail, climbing wall of wire cage and response to vibrissae touch. The maximum score was 15 in absence of deficits.

Rats performed the adhesive removal task (ART) in reversed day-night cycle to allow testing the animals in their active phase. For ART, stickers (Tough-Spots, Diversified Biotech, Boston, MA) were placed on the left and right forepaw and the latency to sticker removal was recorded. The mean time until complete removal of three stickers per forepaw was recorded. Sticker placement on left and right forepaw was alternated between and within animals. The ART was performed under red-light conditions by a trained observer blinded to treatment.

Sensory function was measured using von Frey hairs [19]. Animals were placed on a wire grid bottom through which the von Frey hairs were applied (bending force range from 1 to 15 g; Stoelting, Wood Dale, IL). The hair force was increased or decreased according to response of the animal. Clear paw withdrawal, shaking or licking were considered as increases in mechanical sensitivity. The 50% paw withdrawal threshold was calculated using the up-and-down method [20].

### Statistical Analysis

Data were analyzed by one- or two-way ANOVA with Kruskal Wallis or Bonferroni post-hoc tests. Data are presented as mean ±SEM or boxplot with median and minimal/maximal whiskers.

## Results

### Mortality, acute neurological function and extent of hemorrhage after SAH

**Figure 1:**
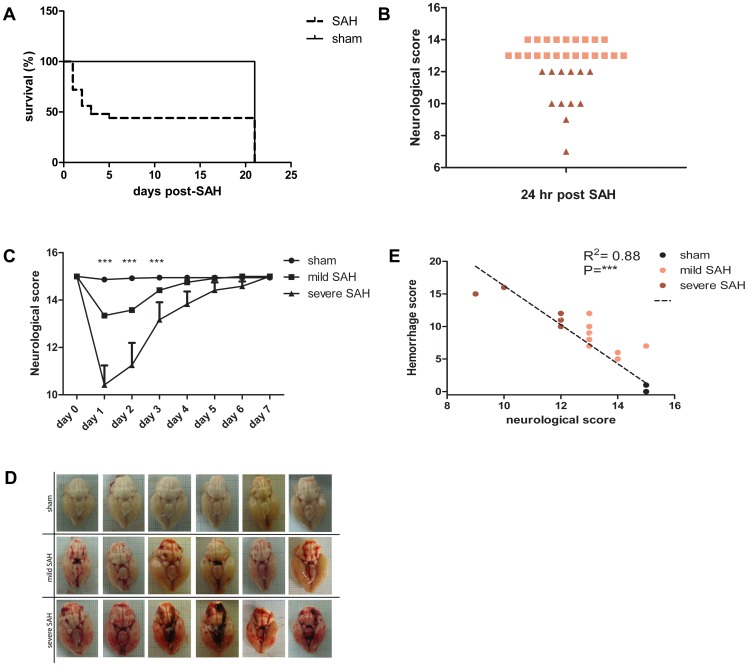
Survival, neurological scores and severity of hemorrhage after SAH. **A**: Survival curve from day 1–21 after induction of SAH. Survival rate is shown for all animals used in this study, except for the rats that were terminated at 48 h post-SAH. Sham n = 19; SAH n = 40 **B**: SAH induces a variety of neurological scores at 24 h after induction of the bleeding. The lowest tertile with neurological scores of 12 or lower was designated as “severe SAH” (red triangles; n = 12). The upper 2 tertiles with minor reductions in neurological scores (14 or 13) were designated as “mild SAH” (pink squares; n = 22). Neurological scores for sham animals were 15 for all animals; n = 33 (data not shown) **C**: Acute neurological scores from day 1–7 after SAH are displayed for all rats that survived for 3 weeks (n = 19 sham; n = 13 mild SAH; n = 6 severe SAH). Stratification in mild or severe SAH was based on neurological scores at 24 h as depicted in Figure 1B. ***p<0.001 vs sham. **D**: Representative photographs from the base of the brain showing the grade of hemorrhage at 48 h after endovascular puncture in all experimental groups. **E**: Correlation between severity of hemorrhage at 48 h in post-mortem brain and acute neurological score at 24 h. *** p<0.001 vs sham. n = 13 (sham; only 2 dots are visible as other values overlap), n = 8 (mild SAH), n = 6 (severe SAH). Data represent mean ±SEM.

SAH was induced by perforating the middle cerebral artery at the level of the circle of Willis. Eleven animals died within 24 h post-SAH (19%), 7 animals died within 48 h post-SAH (12%) and 3 animals died after 48 hours (5,2%) (Figure 1A). All animals died as result of the SAH or when reaching the humane endpoint, except one animal that died of severe bleeding of the carotid artery after surgery. Sham-operation did not lead to mortality.

The neurological score of surviving animals was assessed daily from day 1–7 to determine the acute consequences of SAH. Sham-operated animals did not exhibit any functional deficits in the first days (neurological score 15). At 24 h after induction of the subarachnoid bleeding, we observed a clear variation in the neurological scores of SAH animals, varying from minor reductions in neurological scores (13–14) to more severe reductions in neurological scores (7–12) (Figure 1B). Based on this variety, we designated the lowest tertile of SAH animals as ‘severe SAH’; this group of SAH animals scored ≤12. The animals in the upper tertiles (neurological scores of 13 or 14) were designated as ‘mild SAH’.

Figure 1C shows that both the mildly and severely affected SAH animals showed significantly lower neurological scores at days 1–3 after SAH when compared to sham-operated rats. The neurological score in SAH animals recovered to sham-level from day 3 on (Figure 1C). We also determined the extent of cerebral bleeding by taking post-mortem photographs of the base of the brain at 48 h post-SAH (Figure 1D). These photographs clearly show blood accumulation in the subarachnoid space at 48 h post-SAH. Importantly, the grade of the hemorrhage is clearly related to the severity of the SAH determined by the neurological score at 24 h (Figure 1E). Figure 1E shows the strong correlation (R^2^:0.88) between hemorrhage size and the acute neurological score.

### SAH-induced neuroinflammation in the brain

**Figure 2:**
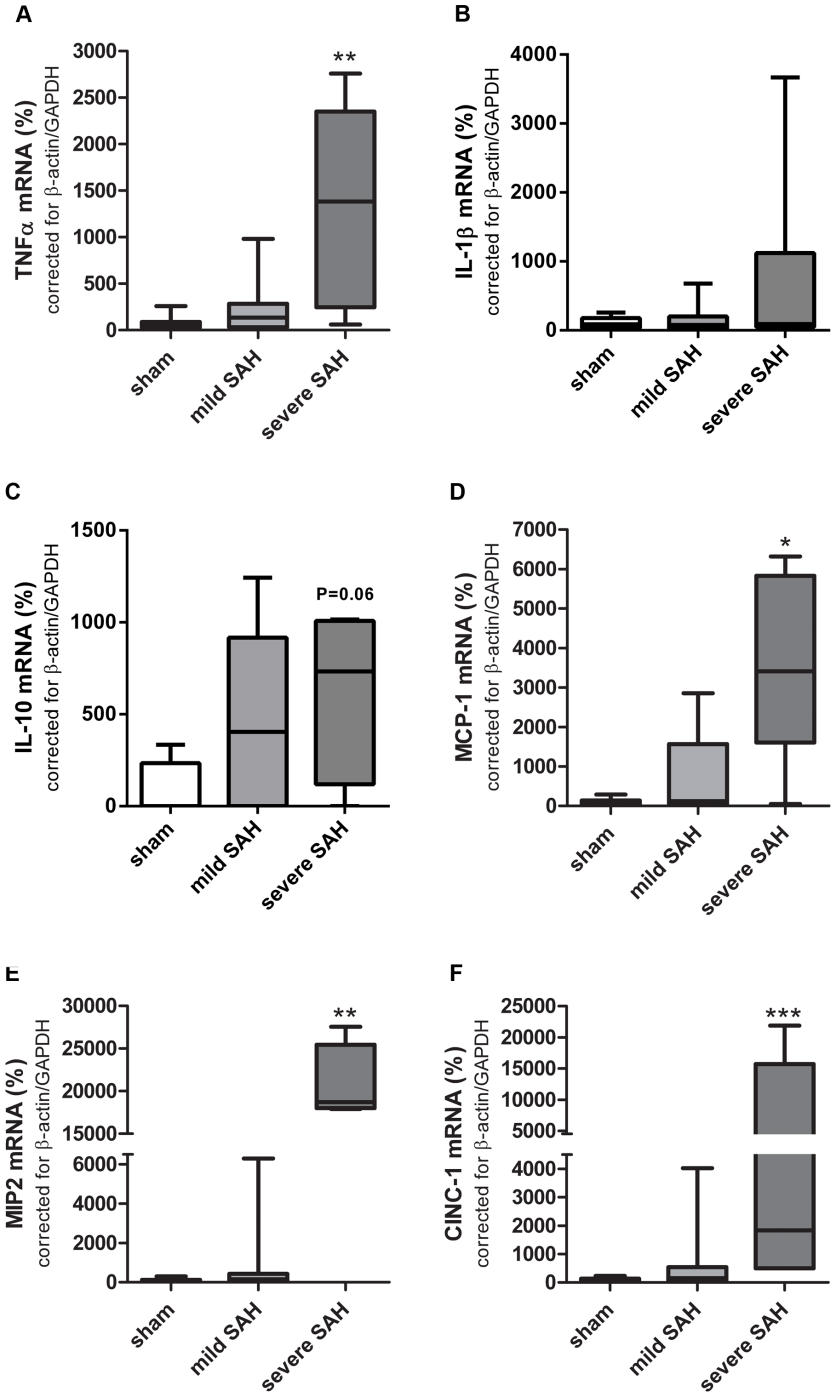
SAH-induced cortical cytokine/chemokine mRNA expression. **A**–**D**: mRNA expression of TNFα (**A**), IL-1β (**B**), IL-10 (**C**), MCP-1 (**D**), MIP2 (**E**) and CINC-1 (**F**) in the cortex at 48 h post-SAH. *p<0.05, **p<0.01, *** p<0.001 vs sham. Data are presented compared to mRNA levels in sham-operated animals which were put at 100%. Sham n = 14; mild-SAH n = 8, severe-SAH n = 6. Data are presented as boxplots with median and minimal/maximal whiskers.

To assess whether the initial neurological scores and hemorrhage grade in mild SAH and severe SAH animals are accompanied by neuroinflammation, we measured mRNA expression of several cytokines and chemokines in the cortex at 48 h after induction of SAH. We selected TNF-α and IL-1β as these cytokines are known to be the most important pro-inflammatory cytokines in human SAH pathology and in other experimental models of SAH (autologous blood injection models) [10], [21]–[24]. IL-10 was selected as an anti-inflammatory cytokine and has shown to be present in serum of SAH patients[25]. TNFα mRNA expression was significantly increased after severe SAH compared to sham-operated animals and mild-SAH animals at 48 h post-SAH (Figure 2A). IL-1β mRNA expression was not significantly increased, which was mainly caused by a large variation in expression levels between animals (Figure 2B). Furthermore, we also measured the anti-inflammatory cytokine IL-10, which showed a trend (p = 0.064) towards increased expression after severe SAH compared to sham-operated animals (Figure 2C).

Furthermore, it has been described that macrophages and neutrophils are the most important immune cells that enter the subarachnoidal space early after SAH [26]. Migration and activation of neutrophils, monocytes and macrophages/microglia in the brain contribute to the neuroinflammatory response after SAH. To investigate the expression of chemotactic factors after SAH we measured MCP-1, MIP2, CINC-1 mRNA expression, which are chemokines well-known to be involved in macrophage and neutrophil recruitment to the brain [27]–[29].

Gene expression of the chemokines MCP-1, CINC-1 and MIP2 was significantly increased after severe SAH compared to sham-operated animals and mild-SAH animals at 48 h post-SAH (Figure 2D–F). Cortical cytokine and chemokine mRNA expression was not significantly increased by mild SAH in comparison to levels in sham-operated rats (Figure 2A–F).

**Figure 3:**
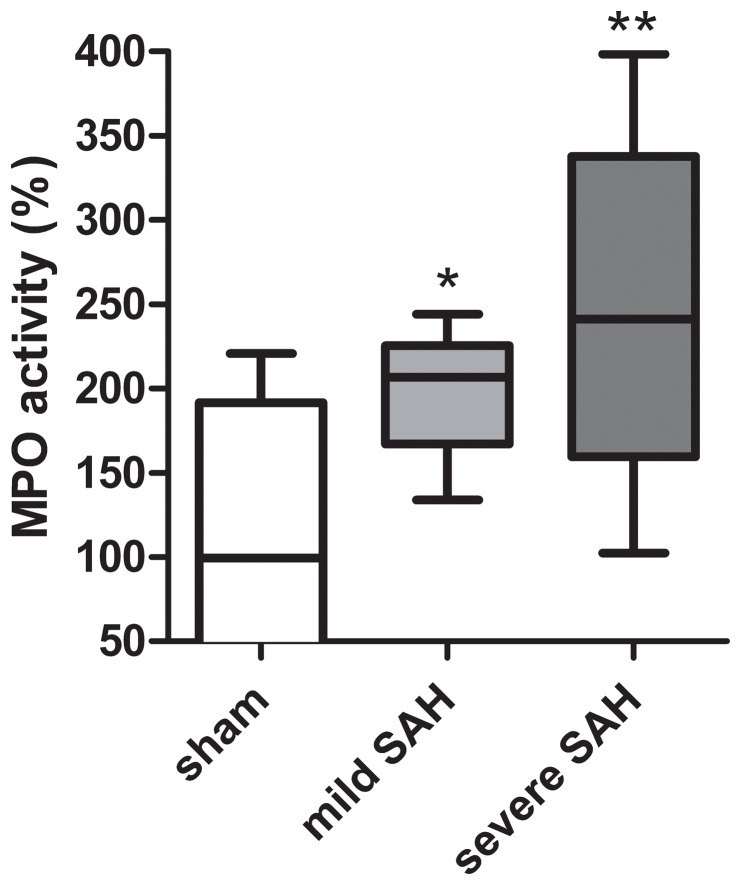
Neutrophil influx at 48 h post-SAH. Cerebral neutrophil influx determined by a MPO assay in the cortex at 48-SAH. Data are presented compared to MPO levels in sham-operated animals which were put at 100%. *p<0.05, **p<0.01 vs sham. Sham n = 14; mild-SAH n = 8, severe-SAH n = 6. Data are presented as a boxplot with median and minimal/maximal whiskers.

**Figure 4:**
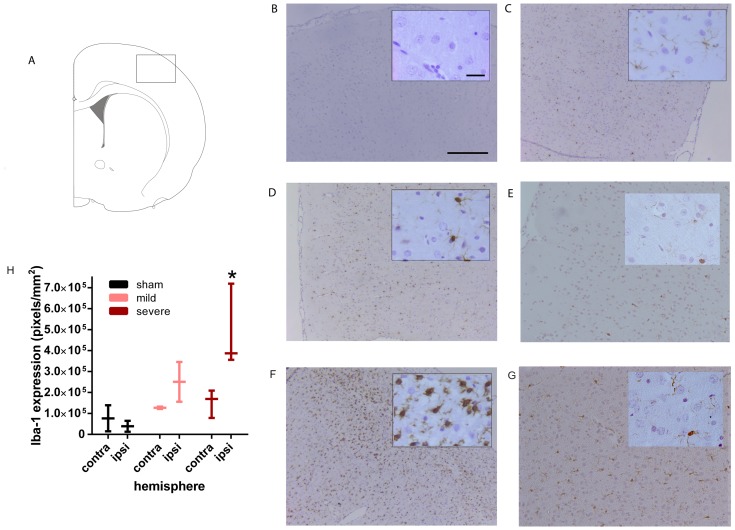
Long-term macrophage/microglia activation after SAH. **A**: Schematic coronal brain view showing where the photographs were taken in the cortex. Photographs are taken at 21 days post-SAH. **B**–**G**: Representative photographs of macrophage/microglia activation by Iba-1 staining in the ipsilateral hemisphere of sham-operated (C), mildly affected SAH (D), severely affected SAH (F) animals and the contralateral hemisphere of a mildly affected SAH animal (E) and severely affected SAH animal (G). B shows a negative control (NC) of the severely affected SAH animal used in F is which the primary antibody was omitted. Insets show a higher magnification. **H**: Quantification of the number of Iba-1 positive pixels in the contralateral and ipsilateral cortex of sham-operated (black), mildly affected SAH (pink) and severely affected SAH (red) animals. Data are presented as a boxplot with median and minimal/maximal whiskers *p<0.05. Scale bar represents 300 µm in the low magnification photograph and 30 µm in the inset.

**Figure 5:**
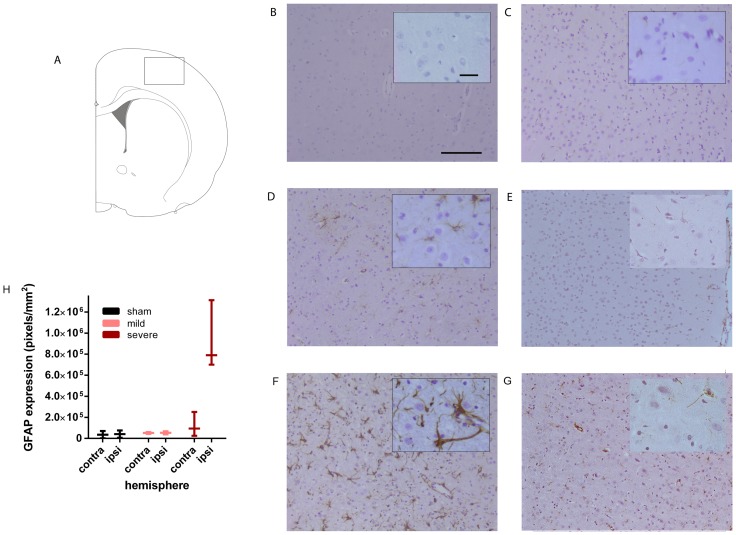
Long-term astrocyte activation after SAH. **A**: Schematic coronal brain view showing where the photographs were taken in the cortex. Photographs are taken at 21 days post-SAH. **B**–**G**: Representative photographs of astrocyte activation by GFAP staining in the ipsilateral hemisphere of sham-operated (C), mildly affected SAH (D) and severely affected SAH (F) animals and the contralateral hemisphere of a mildly affected SAH (E) and severely affected SAH animal(G). B shows a negative control (NC) of the severely affected SAH animal used in F is which the primary antibody was omitted. Insets show a higher magnification. **H**: Quantification of the number of GFAP positive pixels in the contralateral and ipsilateral cortex of sham-operated (black), mildly affected SAH (pink) and severely affected SAH (red) animals. Data are presented as a boxplot with median and minimal/maximal whiskers. Scale bar represents 300 µm in the low magnification photograph and 30 µm in the inset.

Since SAH-induced chemokine expression might result in attraction of immune cells to the lesion site, we determined myeloperoxidase activity (MPO) as a quantitative readout for granulocyte influx at 48 h post-SAH. The number of granulocytes has been shown to be linearly related to the level of MPO [30], although monocytes express a low level of MPO activity as well, which is lost when monocytes differentiate into tissue macrophages [31]–[33]. Cortical MPO activity was significantly increased after both mild and severe SAH compared to sham-controls (Figure 3). These results indicate that SAH induces a recruitment of neutrophils to the cortex. Additionally, we determined whether SAH resulted in a persistent inflammatory state of the brain. At 21 days post-SAH, the presence of activated microglia/macrophages and astrocytes within the cortex (Figure 4A and 5A) was determined using immunohistochemical analysis of Iba-1 and GFAP respectively.

In animals with severe SAH, the number of activated microglia/macrophages, represented by increased Iba-1staining and change in morphology by retraction of cellular branches, was increased in the M1, M2 and S1 layer of the cortex 21 days post-SAH in comparison to sham-operated rats (Figure 4B–G). In animals with mild SAH, a clear increase in Iba-1 staining was visible in the cortex, albeit to a lesser extent than in severely-affected SAH rats (Figure 4B-G). Quantification of Iba-1positivity in the ipsilateral cortex shows a significant increase in Iba-1 in severe SAH animals (Figure 4H). Iba-1 positivity in the contralateral cortex of mild SAH animals did not differ from sham-operated rats, whereas Iba-1 activity was slightly increased in the contralateral cortex of severe SAH animals when compared to sham level (Figure 4E and G).

GFAP staining was slightly increased in mildly-affected SAH rats and activated astrocytes were observed in the M1, M2 and S1 layer of the ipsilateral cortex 21 days post-SAH (Figure 5B–G). Astrocyte activation was strongly increased after severe SAH in the ipsilateral cortex (Figure 5B–G). The increase of GFAP expression after severe SAH was determined by quantification of GFAP positivity in the ipsilateral cortex (Figure 5H). The quantification of GFAP positivity showed that there was a trend (p = 0.09) towards a significant difference between severely SAH affected animals and sham-operated animals. In the contralateral cortex of mild and severe SAH animals, GFAP positivity was similar to GFAP staining in brains of sham-operated animals (Figure 5E and G).

These data together indicate that the degree of long-term neuroinflammation at 21 days was strongly associated with the acute neurological score at 24 h post-SAH and the hemorrhage grade at 48 h post-HI (Figures? 1B–D).

### Long-term neuronal damage after SAH

**Figure 6:**
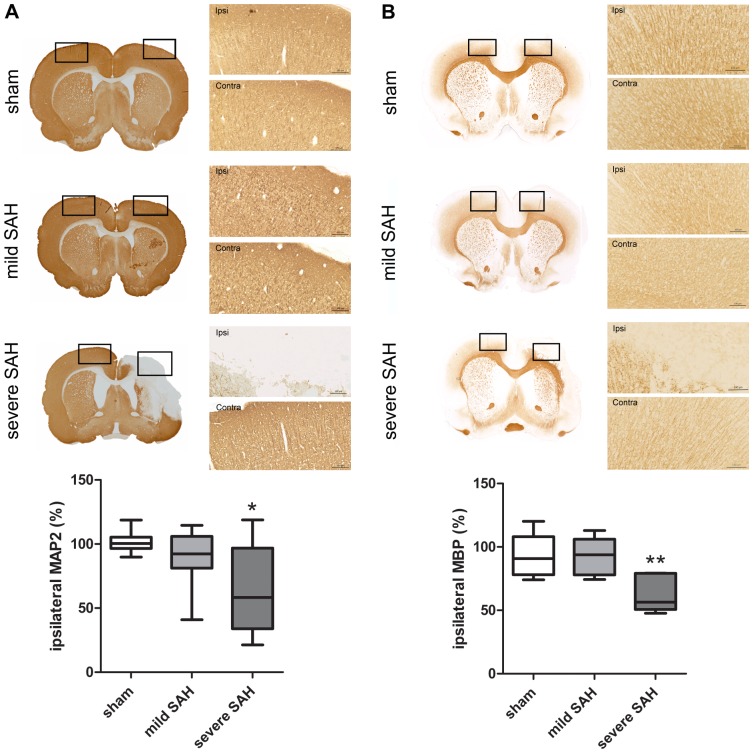
Long-term gray and white matter loss after SAH. **A**: Neuronal damage at 21 days after SAH. Representative photographs of MAP2 loss in the ipsilateral (ipsi) and contralateral (contra) cortex of sham-operated, mildly affected SAH and severely affected SAH animals. Box plot shows ipsi-/contralateral MAP2-positive area*100. *p<0.05 vs sham-operated animals. Data are presented with median and minimal/maximal whiskers. **B**: White matter damage at 21 days after SAH. Representative photographs of MBP loss in the ipsilateral (ipsi) and contralateral (contra) cortex of sham-operated, mildly affected SAH and severely affected SAH animals. Box plot shows ipsi-/contralateral MBP-positive area *100%. **p<0.01. Data are presented with median and minimal/maximal whiskers. **A/B**: Sham n = 19; mild-SAH n = 13, severe-SAH n = 6.

To assess long-term gray matter damage, loss of MAP2 staining was analyzed at day 21. MAP2 loss can be clearly observed in severely-affected SAH animals, especially in the ipsilateral cortex and partially in the striatum, whereas in mildly-affected SAH animals no difference in MAP2 loss was observed compared to sham-operated animals (Figure 6A). No MAP2 loss was observed in sham-operated rats. Injury to the white matter was analyzed by measuring MBP staining at 21 days post-SAH. Animals with severe SAH showed significant MBP loss. There was no significant loss of MBP staining in mildly-affected SAH animals compared to sham-operated animals (Figure 6C).

### Sensorimotor function after SAH

**Figure 7:**
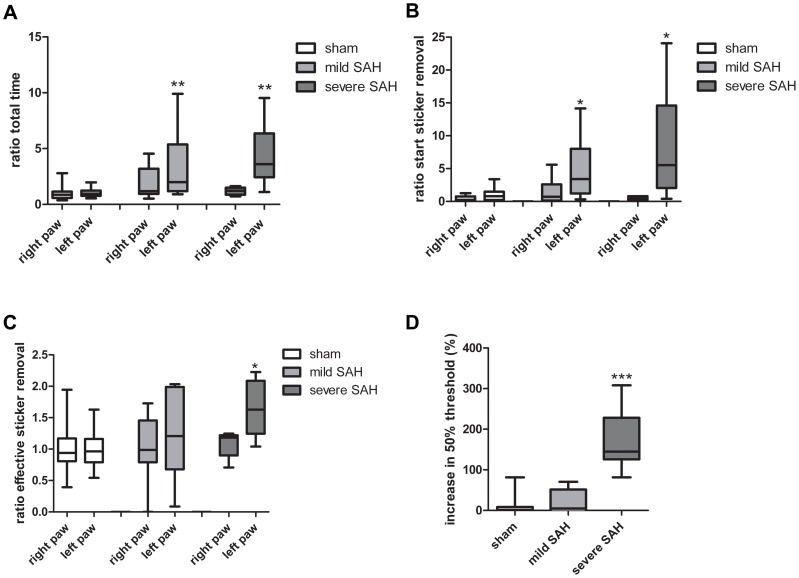
Long-term sensorimotor behavior deficits after SAH. **A**–**C**: Sensorimotor function was tested using the adhesive removal task (ART) at 21d post-SAH. Data are presented compared to sham-operated right paw time measurements which were put at 1. **A**: Total time for adhesive removal. **B**: Latency to start sticker removal (sensory input). **C**: Effective sticker removal time (motor behavior). *p<0.05, ** p<0.01 vs sham. **D**: Mechanical sensitivity was determined using the von Frey test at 21d post-SAH. 50% threshold was determined using von Frey hairs. Sham-operated animals were set at 0% *** p<0.001 vs sham. Data are presented as boxplots with median and minimal/maximal whiskers. **A**–**D**: Sham n = 19; mild-SAH n = 13, severe-SAH n = 6.

At 21 days post-SAH all rats were subjected to the adhesive removal task (ART) in which fine sensorimotor function and forelimb coordination are assessed. Both mildly and severely affected SAH rats needed more time to remove the adhesive from the left (impaired) forepaw when compared to sham-operated rats (Figure 7A). Notably, total time for adhesive removal from the right (unimpaired) forepaw was similar in all groups. To discriminate between sensory and motor function, we measured the time it takes the animals to commence with sticker removal (sensory function; “latency to start removal”) and the time it takes the animals to remove the sticker after sensing it (motor function; “effective sticker removal time”). Both “latency to start removal” and “effective sticker removal time” were significantly increased in severe SAH animals. However, in mild SAH animals only the “latency to start removal” was significantly increased compared to sham-operated animals (Figure 7B–C).

To further explore possible sensory deficits, we tested the sensitivity to touch using the von Frey test 21 days post-SAH[14]. Mechanical sensitivity to innocuous stimuli was significantly decreased in animals with a severe SAH compared to sham-operated animals, indicating a loss of sensory function (Figure 7D). Animals with a mild SAH showed no change in the threshold to perceive the pressure of the von Frey hairs.

## Discussion

In this study we demonstrated, by using the endovascular puncture model in rats, that SAH induces acute neurological dysfunction and long-term brain damage as witnessed by decreased MAP2 and MBP expression. Long-term damage is accompanied by functional deficits in sensorimotor behavior at 21 days post-SAH. Furthermore, SAH induces neuroinflammation characterized by increased cytokine and chemokine mRNA expression and neutrophil influx into the cortex at 48 h after SAH. The neuroinflammatory state of the brain after SAH persisted at least 21 days, as activated microglia/macrophages and astrocytes were still observed at this time-point. The grade of hemorrhage at 48 h and long-term brain damage at 21 days correlated well with the acute neurological score at 24 h post-SAH. Additionally, long-term neuroinflammation was also strongly associated with the severity of SAH assessed by the acute neurological score.

The endovascular puncture model closely resembles the situation in humans suffering from SAH with respect to initial cause of the hemorrhage and physiological parameters [2], [15].

The induction of cerebral inflammation at 48 h after severe SAH is represented by increased TNFα MCP-1, MIP2 and CINC-1 mRNA expression in the cortex. These data are in line with other publications reporting increased pro-inflammatory cytokine and chemokine expression at mRNA and protein level in plasma, CSF and brain tissue at 24 h after SAH [34]–[36]. After cerebral stroke both TNFα and IL-1β protein levels are increased [37]. Our data show that IL-1β mRNA expression was not significantly increased in the cortex after SAH (Figure 2B). These data together indicate a more pivotal role for TNFα than IL-1β in SAH-induced brain damage at 48 h post-insult. Additionally, MCP-1, a chemokine secreted by macrophages/microglia and neurons, that is involved in attracting inflammatory cells like monocytes, is increased after SAH. This is in line with MCP-1 expression found in CSF of human patients after SAH [35]. Moreover, an increase in cerebral MPO activity indicative of increased influx of polymorphonuclear leukocytes into the brain was demonstrated. As MPO activity was determined in non-perfused cortices, both circulating leukocytes in the cortical vasculature as well as leukocyte extravasation into the cortical parenchyma were captured. Both intra-and extravascular leukocytes will contribute to the inflammatory state of the brain and will reflect recruitment and influx of immune cells towards the site of injury. Cerebral inflammation detected at 48 hours after SAH seems to persist since Tiebosch et al. (2013) showed an increased MPO expression even 7 days after SAH using the same endovascular puncture model [28]. Only a few reports about the expression of anti-inflammatory cytokines after SAH are available [25], [38]–[40]. Our results show a trend towards an increased mRNA expression of IL-10 at 48 h post-SAH. In contrast, Aihara et al. (2001) showed no changes in IL-10 expression for several days post-SAH. The role of anti-inflammatory cytokines and their contribution to SAH brain damage remains inconclusive at this time [41].

We suggest that the initial brain damage after SAH results in ongoing inflammation, which may therefore contribute to delayed cerebral ischemia. The use of isoflurane has been shown to significantly influence inflammation [42]. However, Wu et al. (2012) have shown increased inflammation after prolonged exposure (>2 hrs) to isoflurane anesthesia [42]. In this study rats, both SAH and sham-operated, were never exposed to isoflurane for more than 45 minutes. Sham animals have very low levels of TNFα, IL-1β, MCP-1, MIP2 and CINC-1, whereas they underwent similar time period of isoflurane anesthetics. Also in these sham-operated rats MPO activity is significantly less than after SAH.

Our data show for the first time that the neuroinflammation persists for weeks as we show an significantly increased expression and a more active phenotype of microglia/macrophages and astrocytes accumulating at the site of brain damage after severe SAH at 21 days post-SAH. After mild SAH we also observed an increase in Iba-1 and GFAP staining intensity, albeit less than after severe SAH. These data indicate that the long-term neuroinflammatory state of the brain also strongly associates with the acute neurological score measured at 24 h post-SAH. Activated astrocytes are key players in glial scar formation. The role of the glial scar is under debate and is thought to play a protective role in the acute phase after brain damage, partly by demarcating damaged tissue from healthy tissue and thereby preventing spreading of damage signals throughout the brain [43], [44]. However, ongoing activation of astrocytes and scar formation might inhibit axonal outgrowth and remyelination thereby preventing repair processes [43], [44]. Other studies have shown the presence of activated astrocytes after SAH previously [45], [46]. Surprisingly these studies demonstrated astrocyte activation only in the acute phase after SAH (within 1 week) in combination with increased astrocyte/TUNEL co-localization, indicating the death of astrocytes. These data are not in line with our data. A possible explanation might be that in our model the migration of astrocytes to the site of damage outnumbers the overall cell death of astrocytes. However, more research is necessary to test this hypothesis.

SAH also induced a strong activation of Iba-1 within the damaged area at 21 days after induction of SAH. Since Iba-1 is expressed in both microglia and macrophages it is plausible that both resident microglia in the brain are activated after SAH as well as that peripheral macrophages are recruited to the brain after SAH. Activated microglia/macrophages secrete a wide range of factors, including factors that can actively trigger apoptosis like glutamate, TNFα and reactive oxygen species. Microglial/macrophage signaling might also increase astrocyte cytotoxicity, which in turn may cause damage to surrounding cells or lead to changes in neuronal functioning [47], [48]. The presence of activated microglia/macrophages within the lesion site even at 21 days after SAH represents a persistent inflammatory response, which may underlie ongoing neuronal damage. Our data show that Iba-1 expression was also slightly increased in the contralateral hemisphere after severe SAH, which indicates that severe SAH might result in a bilateral long-term neuroinflammatory response.

We are the first to show long-term neuronal loss by a neuron-specific (MAP2) staining after SAH in the endovascular puncture model. To our knowledge the only other study in which neuronal damage was visualized >1 week after SAH is the study by Takata et al. (2008) who showed neuronal loss at 5 weeks after SAH in a model of autologous blood injections [6]. These authors observed neuronal loss mainly in the hippocampal CA1 area and to a lesser degree in the cortex, while our present study shows neuronal loss mainly located in the cortex [6]. These apparently contradictive results in location of SAH-brain injury could be caused by the differences in experimental model. Interestingly, the degree of MAP2 loss at 21 days correlates well with the acute neurological score directly after surgery. The latter indicates that the initial neurological score represents an adequate predictive value for developing long-term brain damage.

We are also the first to show long-term white matter loss after SAH in our model. However, loss of MBP staining was only observed in severely-affected SAH animals and was not detectable in mildly-affected SAH animals. Even when we analyzed MBP staining at a higher magnification for evaluating axonal integrity on a cellular base, we did not see any abnormalities in MBP staining (data not shown). White matter damage measured as MBP loss was clearly less severe than gray matter damage, measured as MAP2 loss. These data indicate that white matter damage could result as a consequence of neuronal damage.

In line with other studies using the endovascular puncture model, we observed unilateral brain damage after SAH in the hemisphere ipsilateral to the endovascular puncture, which is striking since blood in the subarachnoid space will surround both hemispheres. Only in cases of very severe bleeding some studies have shown involvement of the contralateral hemisphere [5]. It might be speculated that the unilateral brain damage after SAH is due to the experimental procedure, as there is a short period of reduced blood flow in the right ICA and MCA, however we feel that this period is very short and not likely to cause ischemic damage. Furthermore the circle of Willis will prevent a total absence of blood flow and thereby ischemic damage. Moreover, in sham-operated animals, which underwent an identical surgical procedure, we did not observe any brain damage or hemorrhage. These data indicate that the SAH-associated early brain damage is a consequence of the induced bleeding and not of the actual occlusion and clamping of the CA during the surgical procedure. Several events following the initial subarachnoid bleeding might be responsible for the development of unilateral brain damage, like increased intracranial pressure, transient global ischemia, a blood clot obstructing the cerebral vasculature or formation of micro-thrombo-emboli [45], [49], [50]. Importantly, delayed ischemia possibly resulting from vasospasms, is an important secondary process that might contribute to neuronal cell death and the observed long-term brain damage after experimental SAH [25], [49], [50]. Since vasospasms are known to be most prominent near the site of the aneurysm [4], this would be a possible explanation for the unilateral brain damage that is observed. Importantly, also in human patients secondary brain damage often occurs and is a major complication after the SAH. Nevertheless, at this moment it remains a limitation of the endovascular puncture model to pinpoint exactly which mechanism, or possibly a combination of the above mentioned mechanisms, is primarily responsible for the observed unilateral damage after SAH in the endovascular puncture model.

To date, there are only few studies showing functional impairments long-term after SAH in animal models and these studies show hardly any functional impairment [6]. However, patients often show long-term functional impairments [1], [2]. Motor deficits following SAH have previously been determined using the tapered beam walk test, horizontal rung walk test, rotarod and cylinder rearing test [5], [51]. These authors showed that the results using the rotarod indicated long-term motor impairment 21days post-SAH [51]. The other motor tests, however, did not reveal motor deficits induced by SAH [5]. The absence of motor deficits in the tampered beam walk test, horizontal ladder test and cylinder rearing test can be explained by the wide variation in hemorrhage volume. Silasi et al. (2009) pointed out that a lot of animals do not survive the first couple of days [5]. Therefore the majority of animals that survive in their experiments 21 days post-SAH are likely to be animals with mild SAH, explaining the absence of significant differences in the motoric tests. The rotarod was been used by various authors [51], who might have induced a higher rate of severely-affected SAH animals resulting in a significant effect of SAH with respect to the rotarod test. Since the animals described in our manuscript are divided in mildly- and severely-affected SAH, this might account for the significant effect in a sensorimotor task: the adhesive removal task. The apparent discrepancy could also be explained by the difference in the tests used to measure motor function. The ART is a test that is more focused on the fine motoric skills and sensorimotor function, while the rotarod is testing motor function and coordination. Our results show, using the ART, that there is a degree of lateralization in sensorimotor behavior after SAH. The tampered beam walk test and horizontal ladder test are more focused on balance and coordination. The lateralizing deficits measured using the adhesive removal task can be partly explained by the insensitivity of these post-SAH rats to touch (or in other words their ability to feel the sticker) and partly by their inability to remove the adhesive, which indicates impaired fine motor skills. However, our preliminary data show that in our model the cylinder rearing test did not show lateralizing deficits following SAH as was also found by Silasi and Colbourne, 2009 [5]. Dividing SAH animals in mildly-and severely-affected SAH animals before using the tapered beam walk test, horizontal ladder test and rotarod might have helped to pinpoint the precise motoric deficits that are present after SAH.

Our study reveals that fine sensorimotor behavior of the rats, as measured in the ART and the mechanical sensitivity as determined in the von Frey test, is affected in severely-affected SAH animals. Severe SAH appeared to impair fine sensorimotor function, since the animals needed longer to sense the tactile stimulus of the sticker as well as to remove the sticker. Mild SAH only affected sensorimotor function in the ART. However, with respect to mechanical sensitivity which can also be viewed upon as a sensory test, only severely affected SAH animals showed a decrease in tactile sensitivity. The latter may imply that the ART may be more sensitive than the von Frey test with respect to measuring tactile discrimination. The SAH-induced impairment in sensorimotor responses which we report here may also explain in part the effects observed in the balance beam performance task as described by Takata et al., 2008 [6]. In this respect the results on the continuous neuroinflammation at the lesion site are intriguing. We propose that neuroinflammation may contribute in this way to development of the functional impairment. If so, there should be space for anti-inflammatory intervention strategies with a wider therapeutic window to improve quality of life of survivors of SAH.

In conclusion, this study shows that SAH induces long-lasting gray and white matter damage, measured at 21 days post-bleeding. SAH is also accompanied by a chronic inflammatory response in the brain which may lead to ongoing cellular damage, formation of scar tissue and neuronal dysfunction.
